# Phylogenetic Characterization of Phosphatase-Expressing Bacterial Communities in Baltic Sea Sediments

**DOI:** 10.1264/jsme2.ME14074

**Published:** 2015-03-28

**Authors:** Anne K. Steenbergh, Paul L.E. Bodelier, Hans L. Hoogveld, Caroline P. Slomp, Hendrikus J. Laanbroek

**Affiliations:** 1 Netherlands Institute of EcologyWageningenThe Netherlands; 2 Department of Earth Sciences (Geochemistry), Faculty of GeosciencesUtrecht University, UtrechtNetherlands; 3 Institute of Environmental Biology, Science FacultyUtrecht University, UtrechtThe Netherlands

**Keywords:** phosphatase, phosphorus biogeochemistry, eutrophication, flow cytometer, ELF labeling

## Abstract

Phosphate release from sediments hampers the remediation of aquatic systems from a eutrophic state. Microbial phosphatases in sediments release phosphorus during organic matter degradation. Despite the important role of phosphatase-expressing bacteria, the identity of these bacteria in sediments is largely unknown. We herein presented a culture-independent method to phylogenetically characterize phosphatase-expressing bacteria in sediments. We labeled whole-cell extracts of Baltic Sea sediments with an artificial phosphatase substrate and sorted phosphatase-expressing cells with a flow cytometer. Their phylogenetic affiliation was determined by Denaturing Gradient Gel Electrophoresis. The phosphatase-expressing bacterial community coarsely reflected the whole-cell bacterial community, with a similar dominance of *Alphaproteobacteria*.

Benthic phosphorus (P) fluxes are an important factor that determines the availability of P for primary producers in the water layer. Whether P is released or retained in sediments is largely controlled by the activity of benthic prokaryotes (6). Therefore, a full understanding of benthic P cycling is needed in order to mitigate the hypoxic conditions caused by anthropogenic influences in many aquatic systems.

Since the majority of P reaches the sediment in the form of organic matter (14), the remineralization of P is the first step potentially leading to P release to the water column. The liberation of phosphate from organic matter is catalyzed by phosphatases. Although this group of enzymes is expressed by many prokaryotes (7), the prokaryotic groups that express phosphatases in sediments have not yet been identified. Phosphatase-expressing benthic microorganisms have previously been characterized with culture-dependent techniques (*e.g.*, 2, 5). However, these techniques may introduce a bias towards organisms that can adapt easily to the culturing conditions (16), and, thus, may not accurately reflect the phosphatase-expressing organisms in the original sediment.

We herein introduced a culture-independent method for determining the phylogenetic affiliation of phosphatase-expressing bacteria in sediments. We previously performed slurry incubations of Baltic Sea sediments, and showed that phosphatases were likely used to relieve the limitation of microbial activity by utilizable carbon (17). In the present study, we labeled whole-cell extracts of these sediment incubations with an artificial substrate for phosphatase and sorted labeled cells using a flow cytometer. We used a PCR-DGGE (PCR-Denaturing Gradient Gel Electrophoresis) analysis targeting bacterial 16S rRNA genes to determine the phylogenetic affiliation of the sorted cells. The results from this study were then compared to the non-sorted whole-cell bacterial community composition of the incubations (which will be referred to as “non-sorted whole-cell fraction”) determined in a previous study, which focused on the impact of redox conditions on the bacterial community structure of Baltic Sea sediments (18). The present study presents a phylogenetic characterization of phosphatase-expressing bacteria in Baltic Sea sediments.

The sediments used in this study were sampled at four stations in the Baltic Sea (LF1, LF1.5, LF3, and LF5) and were incubated for approximately 80 d at 5.2°C. A triplicate full factorial design was used, in which slurries were either incubated under oxic or anoxic conditions, and with or without amendments with glucose, ammonium, and phosphate (“CNP” and “control”, respectively). After being incubated, whole-cells were separated from the slurries by blending and subsequent density centrifugation. The whole-cell fraction was stained with ELF (ELF^®^97 Endogenous Phosphatase Detection Kit; an artificial substrate for phosphatase; Molecular Probes, Eugene, OR, USA). ELF-labeled cells were sorted from the whole-cell fraction with a modular flow cytometer/cell sorter. DNA was extracted from the sorted cells by three cycles of snap-freezing in liquid nitrogen and subsequent thawing, as described in Steenbergh *et al.* (18). The extracts were dialyzed using membrane filters (Millipore MF 0.025 μm VSWP; Millipore, Darmstadt, Germany) to lower the salt content of the samples. PCR targeting 16S rRNA genes, DGGE, sequencing, and a phylogenetic analysis were performed as described in (18). A full description of these methods is given in the supplemental material.

A molecular analysis of phosphatase-expressing cells that were sorted with a flow cytometer resulted in detectable DGGE bands in more than 80% of the samples. The 31 samples that gave a PCR product of the correct size resulted in a total of 85 re-amplifiable DGGE bands. Although a single bacterium can theoretically produce a detectable product on a DGGE gel with the PCR protocol used, the effective detection limit may be increased by several factors. For example, we chose to use a DNA extraction protocol based on snap-freezing and thawing, which is not the most efficient method for lysing bacterial cells, but helps to prevent contamination of the samples. Suboptimal PCR amplification efficiency due to inhibition by co-extracted compounds may also contribute to a higher detection limit (20), and a PCR-DGGE analysis of complex communities only represents the most abundant community members (8). Taken together, an average of 2.7 bands per positive sample was likely an underestimation of the actual number of bacterial phylotypes expressing phosphatases in the sediment samples. The sequences, BLASTN results (1), SILVA (11), and RDP (Ribosomal Database Project) classifier (21) results are given in supplementary Table S1. Of 2 out of the 85 bands, the closest match in BLASTN was related to bacteria found on the skin of humans (see supplementary Table S1). However, the possibility that the DGGE bands originated from benthic bacteria with identical sequences cannot be excluded.

## Abundance of Gram-positive and Gram-negative phosphatase-expressing bacteria

**Fig. 1:**
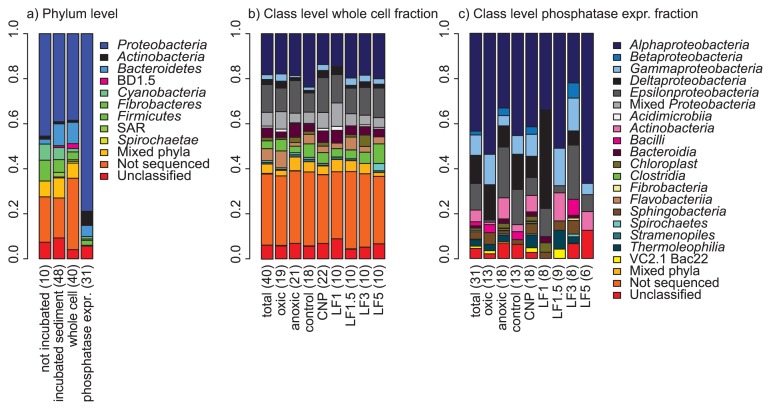
Relative abundance of bacterial phyla (a) and classes (b and c). The numbers between brackets on the x-axes denote the number of samples represented by each bar. (a) Relative abundance of bacterial phyla averaged over sampling stations, treatments, and replicates in the sediments before incubation (“not incubated”); in incubated sediments before the separation of whole cells (“incubated sediment”); in separated whole cells prior to phosphatase labeling and sorting (“whole cell”); in the phosphatase-expressing fraction (“phosphatase expr.”). (b) Relative abundance of bacterial classes averaged across replicates in the whole-cell sample fraction prior to phosphatase labeling and sorting. Abundances were averaged over replicates and over treatments and sampling stations (“total”); over redox conditions during the incubation (“oxic” and “anoxic”); over amendment during the incubation (“control” and “CNP” amendments, in which the latter were amended with carbon, nitrogen, and phosphorus at the start of the incubation); over sampling stations (“LF1”, “LF1.5”, “LF3”, and “LF5”). (c) Relative abundance of bacterial classes in phosphatase-expressing fractions averaged across replicates (where available, see Supplementary material). Data are presented as shown in panel b. Data for all sample fractions, excluding phosphatase-expressing fractions, are adapted from (18; see Supplementary material). “Not sequenced” bacterial taxa represent DGGE bands at retention indices at which no bands were cut for sequencing, whereas “unclassified” taxa were the result of DGGE bands that were sequenced, but were not classified at a ≥95% sequence identity with SILVA (see Supplementary material). Note that the occurrence of “mixed phyla” and “mixed *Proteobacteria*” (*i.e.* DGGE bands at retention indices in gels in which bands from different lanes belonged to more than one phylum or proteobacterial class, respectively) and the higher occurrence of “unknown” taxa in the non-sorted fractions was a result of methodological differences between the present study and reference 18 (see Supplementary material).

The majority of the 85 DGGE bands were affiliated to Gram-negative *Proteobacteria* (70%) and *Bacteroidetes* (7%; Fig. 1 Panel a). Gram-positive bacteria accounted for 12% of the DGGE bands and were represented by *Actinobacteria* (8%) and *Firmicutes* (4%). In the non-sorted whole-cell fraction that was used for flow-cytometric sorting, Gram-positive bacteria accounted for only 5% of the community (adapted from 18). In two studies using culture-dependent methods, a high abundance of Gram-positive phosphatase-expressing bacteria was detected in aquaculture ponds (64%; 2) and in sediment from the Arabian oxygen minimum zone (~45%; 5). In contrast, a culture-independent study of alkaline phosphatase sequences of near-surface planktonic bacteria collected during the Global Ocean Survey (GOS; 7) yielded hardly any phosphatase-coding gene sequences of Gram-positive bacteria, although Gram-positive bacteria were abundant in the GOS samples on the basis of 16S sequences (>12%, 13). This difference in the abundance of Gram-positive phosphatase-expressing bacteria between studies may be attributed to differences in the detection methods used, combined with phosphatase enzymes being expressed at different locations within or outside the cells (4, 7). In Gram-negative bacteria, phosphatases are present in the periplasmic space or in the outer membrane of the cell wall (4). Due to the lack of a periplasmic space in Gram-positive bacteria, a higher percentage of phosphatases expressed by these bacteria may be extracellular. The method used in the present study labeled bacteria that expressed phosphatases as ectocellular enzymes (*i.e.* attached to the outer cell surface or present in the periplasmic space), but not as exoenzymes. In contrast, both types of phosphatase enzymes can be detected using the culture-dependent techniques of the two studies cited above (2, 5), which explains the high abundance of Gram-positive phosphatase-expressing bacteria in these studies. It is currently unclear why few phosphatase-encoding sequences of Gram-positive bacteria were detected in the GOS samples; it may have been due to poor homology between Gram-positive and -negative phosphatase enzymes.

## Class level abundance

Although the number of DGGE bands per sample was lower in the phosphatase-expressing fractions than in the whole-cell fraction before sorting (average of 2.7 vs 9.8 bands per sample, respectively), the relative abundance of bacterial classes was similar for these two sample fractions (Fig. 1; 18). The majority of the DGGE bands of phosphatase-expressing bacteria were affiliated to the *Alphaproteobacteria* (35%). *Betaproteobacteria* were not detected in the non-sorted whole-cell fraction, but accounted for 5% of the phosphatase-expressing bacterial DGGE bands. *Betaproteobacteria* in the GOS samples were also represented more on the basis of phosphatase sequences than on the basis of 16S sequences (approximately 7% compared to 1.7%, respectively; 7, 13). This result suggested that *Betaproteobacteria* made an above average contribution to P liberation. All betaproteobacterial sequences detected in the present study were affiliated to the *Burkholderiales*, and were only present in the CNP-amended incubations. *Burkholderiales* are generally not very abundant in surface sediments, but have been identified as dominant members of bacterial communities in deep sediments (10). The members of this order exhibit high metabolic versatility (*e.g.*, 23), which benefits from the expression of phosphatases. Furthermore, sequences affiliated to the *Actinobacteria*, *Sphingobacteria*, and *Thermoleophilia* classes were absent from the non-sorted whole-cell fraction, but accounted for 5% (*Actinobacteria*) and 3% (*Sphingobacteria* and *Thermoleophilia*) of the DGGE bands in the phosphatase-expressing fraction. This result suggested that, similar to *Betaproteobacteria*, the contribution of these bacterial classes to P cycling in these sediments was higher than was expected based on their abundance.

Although *Gammaproteobacteria* may also be highly abundant in marine sediments (10, 24), only 5% of the sequences of the phosphatase-expressing bacteria were affiliated to this class. The low number of phosphatase-expressing *Gammaproteobacteria* may have been due to the low abundance of *Gammaproteobacteria* in the non-sorted whole-cell fraction (Fig. 1; 18), and not to the low prevalence of phosphatase expression among *Gammaproteobacteria*. In the GOS samples, *Gammaproteobacteria* made a greater contribution to the alkaline phosphatase gene pool than was expected on basis of their abundance in the 16S RNA gene pool, whereas the reverse was true for *Alphaproteobacteria* (7). This was attributed to the higher occurrence of uptake systems for glycerol phosphate (upg) in *Alphaproteobacteria*, which reduced the need for ecto- or extracellular phosphatase enzymes to fulfill their P demands. As the abundance of phosphatase-expressing *Alpha*- and *Gammaproteobacteria* in the present study corresponded with their abundance in the whole-cell fraction, this difference in physiology between the two classes was not detectable at the phosphatase expression level for these Baltic Sea sediments.

*Deltaproteobacteria* made up 2% of the non-sorted whole-cell bacterial community (18), but accounted for 7% of the DGGE bands of the phosphatase-expressing bacteria. Of these six phosphatase-expressing *Deltaproteobacteria*, five were the most closely affiliated to the *Desulfobacterales*. Although sulfate-reducing bacteria are predominantly active under anoxic conditions, their abundance in the phosphatase-expressing fraction was similar under the oxic and anoxic incubations. Sulfate reducers typically only oxidize simple organic fermentation products, but may also utilize more complex compounds, such as hydrocarbons and amino acids (9, 12). However, these compounds do not contain any P and, thus, do not require phosphatase activity. *Deltaproteobacteria* may express phosphatases to meet their P requirements, but pore-water phosphate concentrations are generally high and microbial activity in the Baltic Sea sediments was limited by C, but not P-availability (17). Phosphatase-coding sequences from *Deltaproteobacteria* were detected in the GOS samples, in which P limitation was more likely to occur, although they made up only a small percentage of the metagenomic database (7). Apart from hydrolyzing C-P bonds, phosphatases exhibit the ability to catalyze the thermodynamically favorable oxidation of phosphite (PO_3_^3−^) to phosphate and molecular hydrogen (22). Bacteria capable of phosphite oxidation are abundant in soil and sediment (19), and the bacterium *Desulfotignum phosphitoxidans* can grow using phosphite oxidation as its sole energy source (15). We did not determine phosphite concentrations in our samples. However, since phosphite is unstable, except under low redox conditions, phosphite concentrations in sediments are considered to be very low (15). Therefore, the function of phosphatase activity in phosphatase-expressing *Deltaproteobacteria* in the Baltic Sea sediments remains uncertain.

The abundance of phosphatase-expressing *Epsilonproteobacteria* was higher in the anoxic (16%, *n*=54) than in the oxic (2%, *n*=31) incubations, which was expected because *Epsilonproteobacteria* often occur in sulfidic habitats (3); however, this difference was not significant (*t*-test, *p*=0.10).

In summary, we herein linked the phosphatase activity of Baltic Sea sediments to bacterial phylogenetic taxa using a PCR-DGGE analysis of sorted ELF-labeled cells. The phosphatase-expressing community in these sediments coarsely reflected the non-sorted whole-cell bacterial community; however, a number of bacterial classes were only detected in the phosphatase-expressing sample fractions. The diversity in phosphatase enzymes precludes the use of one single method to phylogenetically characterize all phosphatase-expressing prokaryotes in a community. The method described in this study provided a culture-independent way to characterize bacteria expressing phosphatases as ectoenzymes. More detailed phylogenetic information can be gained using this method when more recently developed sequencing techniques are used instead of PCR-DGGE-based sequencing.

## Supplementary Information
