# Supplementary material for: Phylogenetic Characterization of Phosphatase-Expressing Bacterial Communities in Baltic Sea Sediments
Source: Microbes Environ. 2015 Mar 28;30(2):192–5. doi: 10.1264/jsme2.ME14074 (PMC4462931; doi:10.1264/jsme2.ME14074)
Supplement: Supplementary file 1 [file 30_192_s1.pdf]

Phylogenetic characterization of phosphatase-expressing bacteria in Baltic Sea sediments

Supplemental material: methods

Sediment sampling and incubation

Sediment top layers (0-1 cm) were sampled at four stations in the Baltic Sea (Stations LF1, LF1.5, LF3, and LF5), positioned along a depth transect from 67 to 135 m into the Fårö Deep (see 1) for a detailed description of sites and sediments). Due to their position relative to the oxycline in the Baltic Sea, bottom water oxygen concentrations varied from oxic (station LF1) to anoxic and sulfidic (station LF5). The sediments were incubated as slurries at 5.2 ºC for approximately 80 days. When assuming a specific growth rate of 0.05 d^{-1} at this temperature (5), this would allow for on average 4 division cycles. The slurries were incubated under oxic or anoxic conditions, and with or without amendment with glucose, ammonium, and phosphate ("CNP" or "control", respectively) in a triplicate full factorial experimental design (total of 48 incubations). After incubation, whole-cells were separated from the slurries by blending and subsequent density centrifugation according to the method described by Lindahl (2) with minor modifications. Approximately 6 ml of incubated sediment slurry was brought to a volume of approximately 10 ml with filtered (0.22 μm) 9.34 ‰ NaCl in Milli-Q water in a 50 ml Falcon tube. The resulting slurry was blended 3 times during 1 minute with an Ultra-Turrax blender (IKA-Werke, Stauten, Germany) and cooled in between and afterwards on ice. The slurries were then brought to a volume of approximately 40 ml with the 9.34 ‰ NaCl solution and shaken thoroughly. Larger sediment particles were allowed to settle for 15 minutes. Twenty-nine ml of the suspension was transferred to a 43 ml polycarbonate centrifuge tube (Nalgene, Rochester, NY), and a cushion of 6 ml Nycodenz solution (Axis-Shield PoC AS, Oslo, Norway, 80% w/v in the 9.34 ‰ NaCl solution) was pipetted carefully

underneath. Samples were centrifuged for 1 hour at 10.000 ×g at 4 °C in a Sorvall RC-6 Plus (Thermo Scientific) with a Sorvall HB-6 swing-out rotor (Thermo Scientific). Approximately 27 ml of the top layer was pipetted off and discarded. Three milliliters of sample at the interface between Nycodenz cushion and top layer were transferred to a clean centrifuge tube. These samples were brought to a volume of 29 ml with 9.34 ‰ NaCl solution, and again a 6 ml Nycodenz cushion was pipetted underneath. Samples were centrifuged under the same conditions for 2 hours. The 3 ml at the Nycodenz-water interface was transferred to a clean centrifuge tube and brought to a volume of 35 ml with 9.34 ‰ NaCl solution and vortexed. The cells were pelleted by another hour of centrifugation under the same conditions. Thirty milliliters of the supernatant was pipetted off and discarded. The cells were resuspended in the remaining 5 ml. The whole-cell fraction was stained with ELF (ELF®97 Endogenous Phosphatase Detection Kit, Molecular Probes; an artificial substrate for phosphatase) for 48 h at room temperature. A detailed description of sediment incubation and sample preparation is given in an accompanying paper (8).

Flow-cytometric sorting

The experiments were carried out with a modular flow cytometer/cell sorter, MoFlo® (initially from DakoCytomation, Denmark, now Beckman-Coulter, USA), as described in Steenbergh et al. (8). The instrument was equipped with a Smart Sampler station (an automated oscillating sample probe inside a pressurized chamber) and a CyClone unit, which is a computer-driven robotic table that automatically positions the collection vessel so that sorted events can be deposited according to a predetermined matrix. Data acquisition and analyses were performed with the software package Summit™, version 4.3.01 (DakoCytomation).

Prior to the sorting of every sample, the delay for droplet charging was calibrated with 6 μm Fluoresbrite carboxylate yellow / green (YG) microspheres (Polysciences Inc. Europe, Germany). During the actual sorting, the stability of the jet, droplet formation and position of the break-off point were monitored on-screen with the use of a stroboscopic camera.

Sorting for DNA-extraction was performed on particles in a gate, formed by real events (based on side scatter) and ELF-positive events (UV-laser/530 nm). The gate settings for classifying a particle as ELF-positive were determined as part of a previously published study (8) on sub-samples of each sample of the whole-cell fraction that either were or were not stained with ELF and propidium iodide (i.e., four sub-samples per sample; propidium iodide stains DNA). The data rate was kept below 5000 events per second to avoid coincidence (<1 %). Thirty eight samples - out of a total of 48 available samples - were sorted in this way; 10 samples from each station, except for Station LF5, from which 8 samples were sorted. On average, 4.4 10^5 labeled cells were sorted per sample.

DNA extraction

To reduce the risk of contamination of the samples, we chose to extract DNA from the sorted cells by three cycles of snap-freezing in liquid nitrogen and subsequent thawing, as described in Steenbergh et al. (7). The extracts were dialyzed using membrane filters (Millipore MF 0.025μm VSWP) to lower the salt content of the samples.

PCR-DGGE

Due to low template concentrations, we used a nested PCR scheme targeting 16S rRNA genes (as described by 7). The first step consisted of 35 cycles (annealing temperature 65-55 ºC; 3), for the second step the profile was reduced to the first 25 cycles. The primers for the first step were F27 (5'-AGA GTT TGA TYM TGG CTC AG-3') and 1492R (5'-TAC GGY TAC CTT

GTT ACG ACT-3'), and for the second step GC-F357 (5'-CGC CCG CCG CGC CCC GCG CCC GGC CCG CCG CCC CCG CCC CCC TAC GGG AGG CAG CAG-3') and R518 (5'-ATT ACC GCG GCT GCT GG-3'; all primers: Thermo Fisher Scientific, Ulm, Germany). DGGE, re-amplification of cut bands and sequencing was performed as described in Steenbergh et al (7). The 8% acrylamide gels contained a 20 – 80% denaturing gradient and were run in a Protean II system (Bio-Rad, USA) at 60 ºC, 100 V for 17 h. All bands were cut and reamplified. The amplicons of 'clean' bands (that is, cut bands that resulted in single bands on DGGE after a maximum of 4 cycles of re-amplification and DGGE analysis) were sequenced (Macrogen, Korea). DGGE bands that did not result in clean bands were regarded to be artefacts and were excluded from further analyses.

Phylogenetic analysis

We assigned phylogenetic tags to the sequences as described by Steenbergh et al (7). The software package Sequencher 4.2 (Gene Codes Corporation, Ann Arbor, MI) was used to assemble and inspect contigs. Aligning and filtering were performed with mothur (v.1.19.3) (6). All sequences were submitted to the European Nucleotide Archive (ENA; accession numbers LN774886 - LN774969; see supplementary Table S1). Phylogenetic tags were assigned using the SILVA database (SILVA Incremental Aligner (SINA) Summary version 1.2.11, September 2014) (4) and with the RDP (Ribosomal Database Project) naïve Bayesian rRNA Classifier (Version 2.2, March 2010; 9). Sequences with < 100% score at the domain level or < 95% score at phylum level were left unclassified ("unclassified"). Similarly, sequences with <95% score at the class level were only classified up to phylum level. The number of DGGE bands that were unclassified was lower using the SILVA database (6 at both phylum and class level) compared to the RDP classifier (18 and 25 at phylum and class level, respectively). We therefore chose the results of the SILVA database for further analysis.

Because of the low number of DGGE bands per sample, and the high number of PCR cycles used for template amplification, we chose to score abundances on basis of presence/absence, not on the relative intensity of the DGGE bands.

To compare the data from the phosphatase-expressing fraction to the whole-cell fraction before sorting, we have re-analyzed data from a previous paper (7). Phylogenetic tags were now assigned using the SILVA database and DGGE bands were scored according to presence/absence. Due to the larger number of DGGE bands per sample for the unsorted fraction compared to the phosphatase-expressing fraction, not all DGGE bands were cut and reamplified. All bands at a specific retention index were assigned to the same phylogenetic tag. DGGE bands at retention indices at which no bands were cut for sequencing, were labeled as "not sequenced", and bands at indices that contained multiple phylogenetic tags as "mixed phyla". As a result, the number of DGGE bands with a mixed phylogenetic assignment and not-sequenced bands is higher for this unsorted whole-cell fraction.

References

1. Jilbert T.S., C. P. Slomp, B.G. Gustafsson, and W. Boer. 2011. Beyond the Fe-P-redox connection: preferential regeneration of phosphorus from organic matter as a key control on Baltic Sea nutrient cycles. Biogeosciences Discuss 8: 655-706.

2. Lindahl **V**. Improved soil dispersion procedures for total bacterial counts, extraction of indigenous bacteria and cell survival. 1996. Journal Microbiol Meth 25: 279-286.

3. Muyzer G., E.C. de Waal, and A.G. Uitterlinden. 1993. Profiling of complex microbial populations by denaturing gradient gel electrophoresis analysis of polymerase chain reaction-amplified genes coding for 16S rRNA. Appl Environ Microbiol 59: 695-700.

4. Pruesse E, J. Peplies, and F.O. Glöckner. 2012. SINA: Accurate high-throughput multiple sequence alignment of ribosomal RNA genes. Bioinformatics 28: 1823-1829.

5. Sander B.C., and J. Kalff. 1993. Factors controlling bacterial production in marine and fresh-water sediments. Microbial Ecology 26: 79-99.

6. Schloss P.D., S.L. Westcott, T. Ryabin, ***et al***. 2009. Introducing mothur: Open-Source, Platform-Independent, Community-Supported Software for Describing and Comparing Microbial Communities. Appl Environ Microbiol 75: 7537-7541.

7. Steenbergh A.K., P.L.E. Bodelier, C.P. Slomp, and H.J. Laanbroek. 2014. Effect of Redox Conditions on Bacterial Community Structure in Baltic Sea Sediments with Contrasting Phosphorus Fluxes. Plos One 9: e92401.

8. Steenbergh A.K., P.L.E. Bodelier, H.L. Hoogveld, C.P. Slomp, and H.J. Laanbroek. 2011. Phosphatases relieve carbon limitation of microbial activity in Baltic Sea sediments along a redox-gradient. Limnol Oceanogr 56: 2018-2026.

9. Wang Q., G.M. Garrity, J.M. Tiedje, and J.R. Cole. 2007. Naïve Bayesian Classifier for Rapid Assignment of rRNA Sequences into the New Bacterial Taxonomy. Appl Environ Microbiol 73: 5261-5267.
