# Supplementary material for: Phylogenetic Characterization of Phosphatase-Expressing Bacterial Communities in Baltic Sea Sediments
Source: Microbes Environ. 2015 Mar 28;30(2):192–5. doi: 10.1264/jsme2.ME14074 (PMC4462931; doi:10.1264/jsme2.ME14074)
Supplement: Supplementary file 2 [file 30_192_s4.docx]

Supplementary material: full description of methods.

Supplementary Table 1: Sequences, BLASTN, RDP classifier, and SILVA database results of cut DGGE bands.

Supplementary Table 2: Data of phylum- and class-level abundances, as used for Fig. 1.
